# Crystal structure and Hirshfeld surface analysis of 2,4-di­amino-6-[(1*Z*,3*E*)-1-cyano-2,4-di­phenyl­penta-1,3-dien-1-yl]pyridine-3,5-dicarbo­nitrile monohydrate

**DOI:** 10.1107/S2056989024002962

**Published:** 2024-04-18

**Authors:** İbrahim G. Mamedov, Victor N. Khrustalev, Mehmet Akkurt, Fuad Sh. Kerimli, Ajaya Bhattarai, Ali N. Khalilov, Farid N. Naghiyev

**Affiliations:** a Department of Chemistry, Baku State University, Z. Khalilov str. 23, Az, 1148, Baku, Azerbaijan; b Peoples’ Friendship University of Russia (RUDN University), Miklukho-Maklay St. 6, Moscow, 117198, Russian Federation; c N. D. Zelinsky Institute of Organic Chemistry RAS, Leninsky Prosp. 47, Moscow, 119991, Russian Federation; d Department of Physics, Faculty of Sciences, Erciyes University, 38039 Kayseri, Türkiye; e Department of Chemistry, M.M.A.M.C (Tribhuvan University) Biratnagar, Nepal; f "Composite Materials" Scientific Research Center, Azerbaijan State Economic University (UNEC), H. Aliyev str. 135, Az 1063, Baku, Azerbaijan; University of Hyogo, Japan

**Keywords:** crystal structure, hydrogen bonds, pyridine ring, C—H⋯π inter­actions, Hirshfeld surface analysis

## Abstract

The asymmetric unit of the title compound, C_25_H_18_N_6_·H_2_O, comproses two mol­ecules (**I** and **II**), together with a water mol­ecule. The terminal phenyl groups attached to the methyl groups of the mol­ecules **I** and **II** do not overlap completely, but are approximately perpendicular. In the crystal, the mol­ecules are connected by N—H⋯N, C—H⋯N, O—H⋯N and N—H⋯O hydrogen bonds with each other directly and through water mol­ecules, forming layers parallel to the (001) plane. C—H⋯π inter­actions between these layers ensure the cohesion of the crystal structure. A Hirshfeld surface analysis indicates that H⋯H (39.1% for mol­ecule **I**; 40.0% for mol­ecule **II**), C⋯H/H⋯C (26.6% for mol­ecule **I** and 25.8% for mol­ecule **II**) and N⋯H/H⋯N (24.3% for mol­ecules **I** and **II**) inter­actions are the most important contributors to the crystal packing.

## Abstract 2

In the crystal, mol­ecules are connected by N—H⋯N and C—H⋯N, and O—H⋯N and N–H⋯O hydrogen bonds, to each other directly and through water mol­ecules, forming layers parallel to the (001) plane. C—H⋯π inter­actions between these layers ensure the cohesion of the crystal structure.

## 1. Chemical context

**Figure 1:**
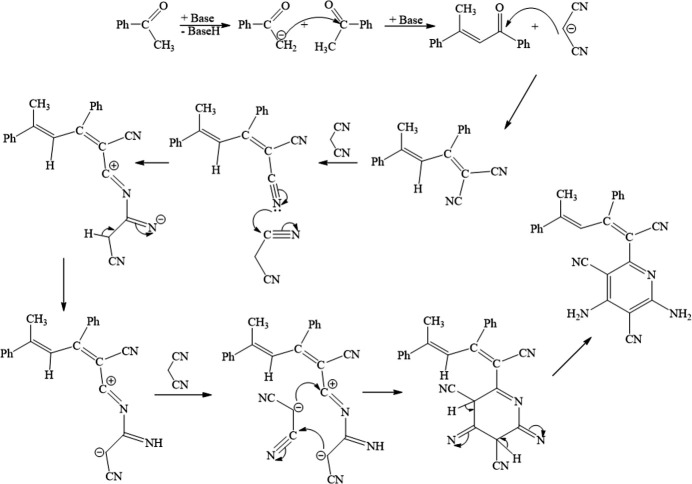
The plausible formation mechanism of the title compound.

Functionalized pyridines are six-membered heterocyclic systems containing one or several functional groups in their core. These derivatives are used for a large range of applications and as as drugs, ligands, catalysts, materials *etc* (Maharramov *et al.*, 2021 ▸; Sobhi & Faisal, 2023 ▸). Functionalized pyridines with various biological activities, such as anti­cancer, anti­oxidant, vasodilatory, cytotoxic, anti-inflammatory, herbicidal, insecticidal, anti­hypertensive, anti­bacterial, anti­convulsant, cardiotonic properties, as well as multiple synthetic pathways of these systems, have been reported (Atalay *et al.*, 2022 ▸; Donmez & Turkyılmaz, 2022 ▸; Abd El-Lateef *et al.*, 2023 ▸). Given the wide application of these compounds, the efficient and regioselective functionalization of pyridines has attracted much attention. Thus, in the framework of our studies in heterocyclic chemistry (Naghiyev *et al.*, 2020 ▸, 2021 ▸, 2022 ▸), herein we report the crystal structure and Hirshfeld surface analysis of the title compound, 2,4-di­amino-6-[(1*Z*,3*E*)-1-cyano-2,4-di­phenyl­penta-1,3-dien-1-yl]pyridine-3,5-dicarbo­nitrile. The plausible reaction mechanism of the formation of the title compound is illustrated in Fig. 1 ▸. 
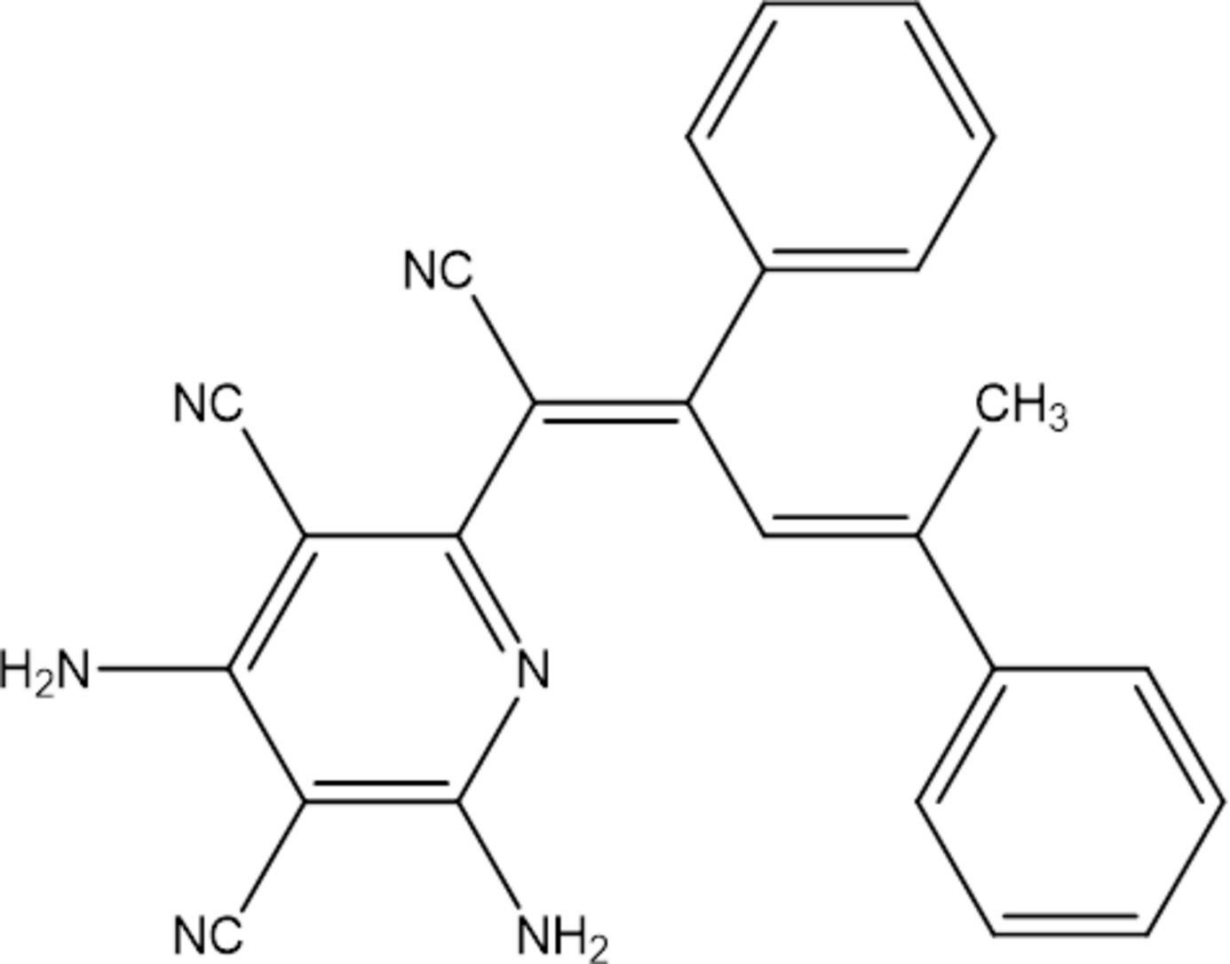


## 2. Structural commentary

**Figure 2:**
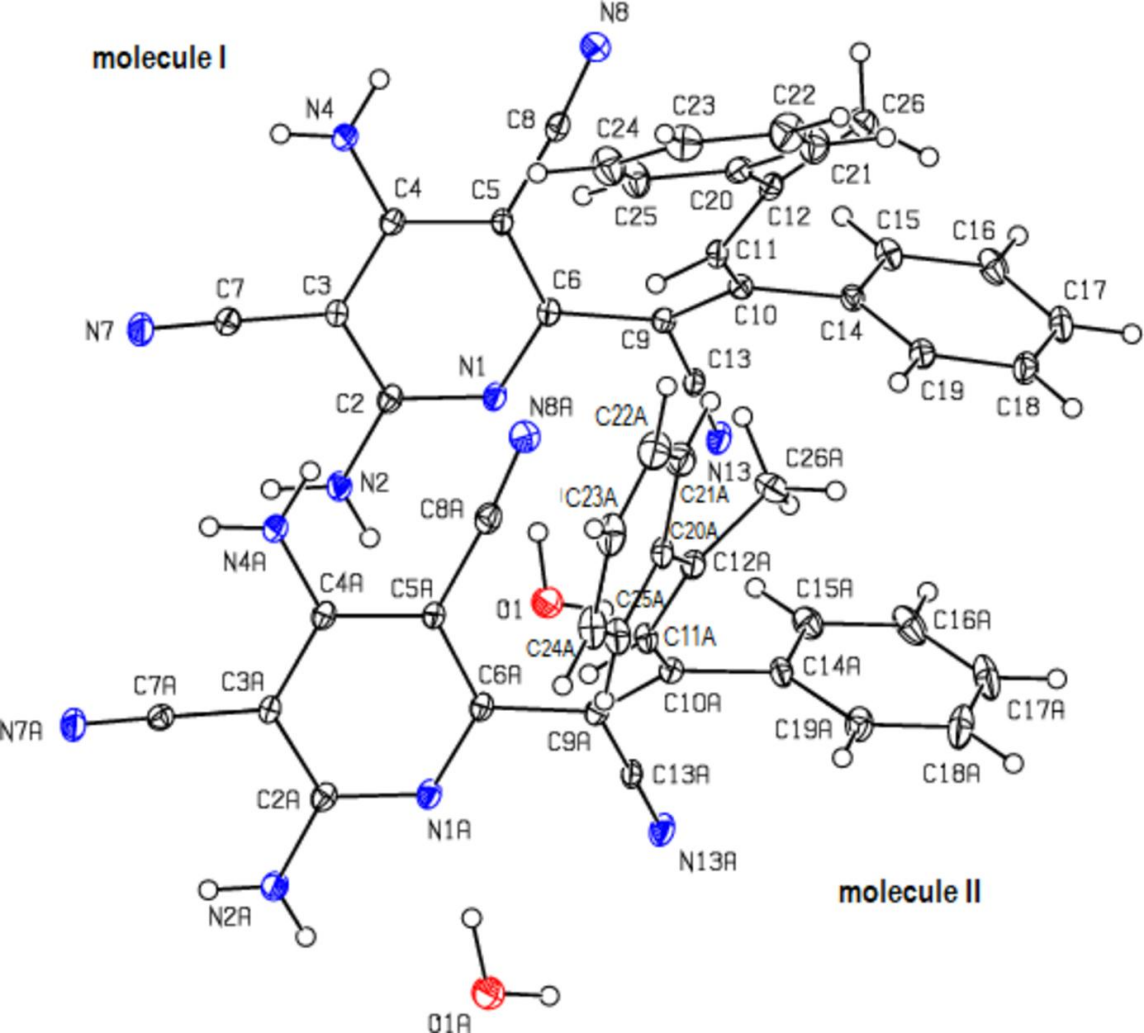
The mol­ecular structure of the title compound, showing the atom labelling and displacement ellipsoids drawn at the 30% probability level.

**Figure 3:**
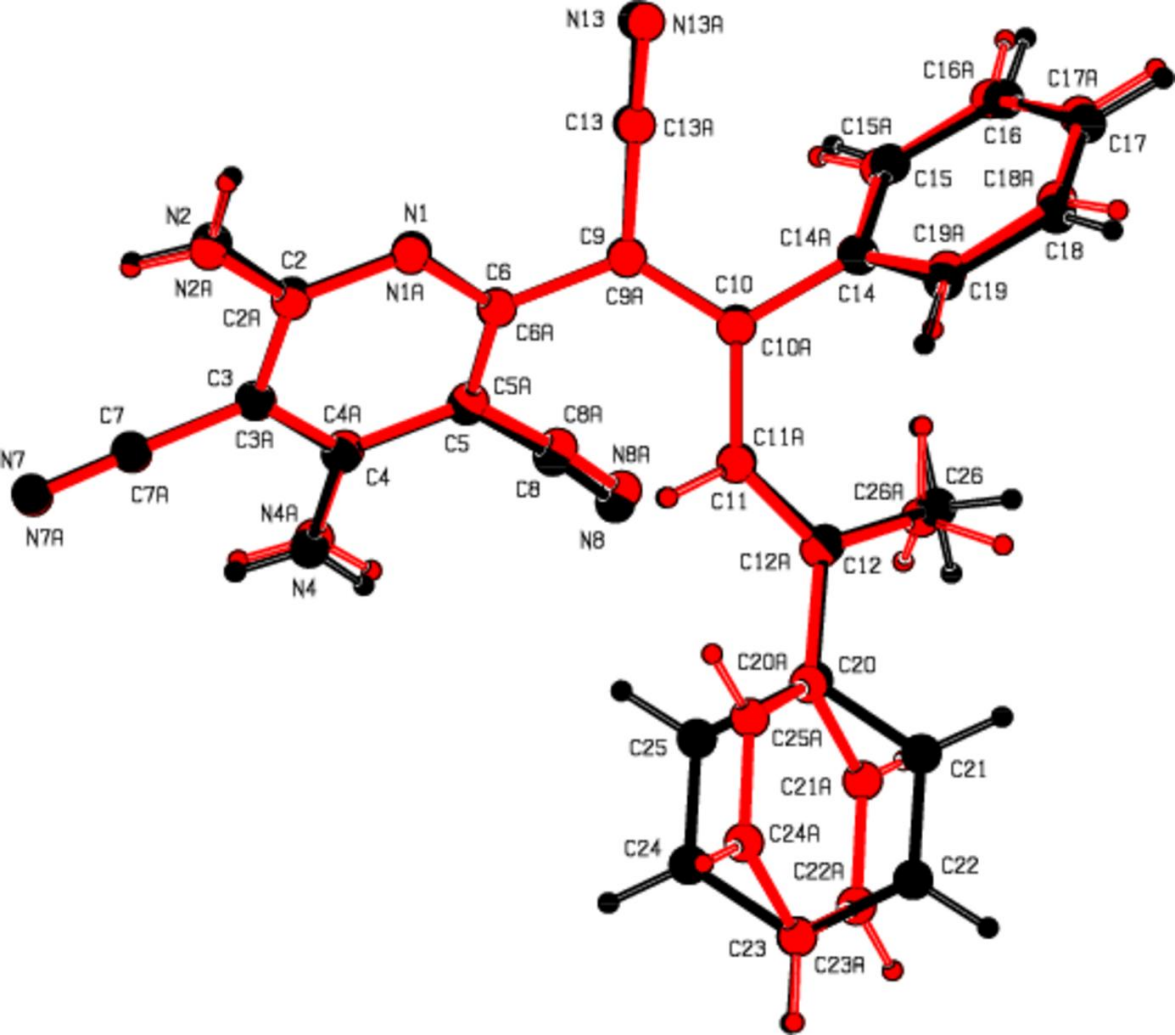
A least-squares overlay of the two independent mol­ecules **I** and **II** [inverted mol­ecule **II** (red) on mol­ecule **I** (black)].

Fig. 2 ▸ shows two mol­ecules (**I** without suffix and **II** with suffix *A*), which together with a water mol­ecule form the asymmetric unit. An overlay fit of inverted mol­ecule **II** on mol­ecule **I** is shown in Fig. 3 ▸, the weighted r.m.s. fit of the 31 non-H atoms being 0.510 Å and showing the major differences to be in the terminal phenyl groups (C20–C25 and C20*A*–C25*A*) attached to the methyl groups of the mol­ecules **I** and **II**.

In **I**, the phenyl rings (C14–C19 and C20–C25) form a di­hedral angle of 45.39 (11)° with each other, while they subtend angles of 80.43 (10) and 57.35 (10)°, respectively, with the pyridine ring (N1/C2–C6). In **II**, the phenyl rings (C14*A*–C19*A* and C20*A*–C25*A*) form a dihedral angle of 87.88 (11)° with each other, while they subtend angles of 76.94 (11) and 62.05 (10)°, respectively, with the pyridine ring (N1*A*/C2*A*–C6*A*). In **I**, the C6—C9—C10—C14, C6—C9—C10—C11, C9—C10—C11—C12 and C10—C11—C12—C20 torsion angles are 177.30 (18), −11.2 (3), 153.8 (2) and 174.73 (19)°, respectively. In **II**, the corresponding C6*A*—C9*A*—C10*A*—C14*A*, C6*A*—C9*A*—C10*A*—C11*A*, C9*A*—C10*A*—C11*A*—C12*A* and C10*A*—C11*A*—C12*A*—C20*A* torsion angles have approximately the same values, *viz*. 172.10 (19), −15.5 (3), 153.0 (2) and 173.0 (2)°, respectively. Bond lengths and angles in the mol­ecules of the title compound are comparable with those of closely related structures detailed in the *Database survey* (section 4).

## 3. Supra­molecular features and Hirshfeld surface analysis

**Figure 4:**
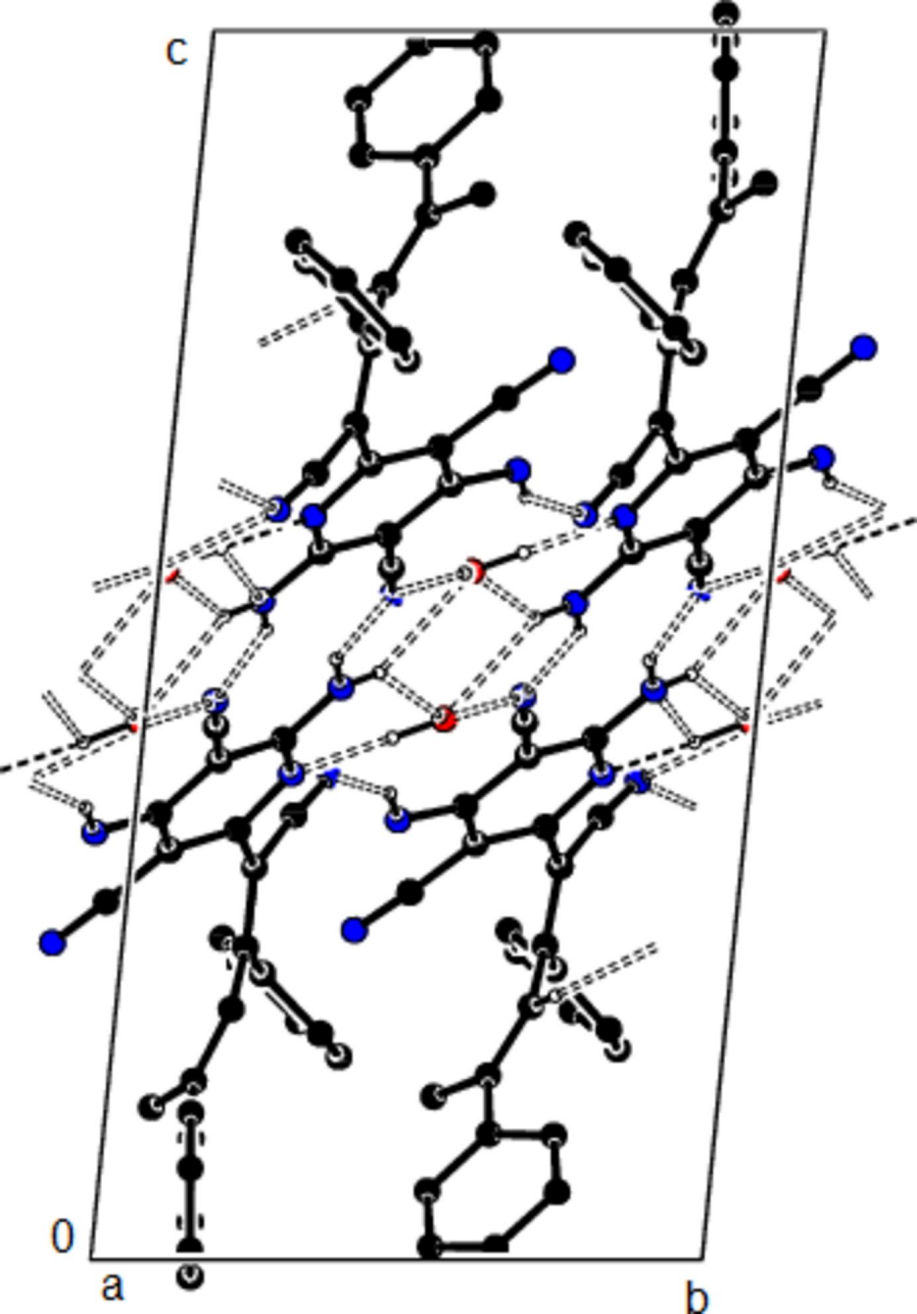
The packing of the title compound viewed along the *a* axis with O—H⋯N, N—H⋯O, N—H⋯N and C—H⋯N hydrogen bonds shown as dashed lines.

**Figure 7:**
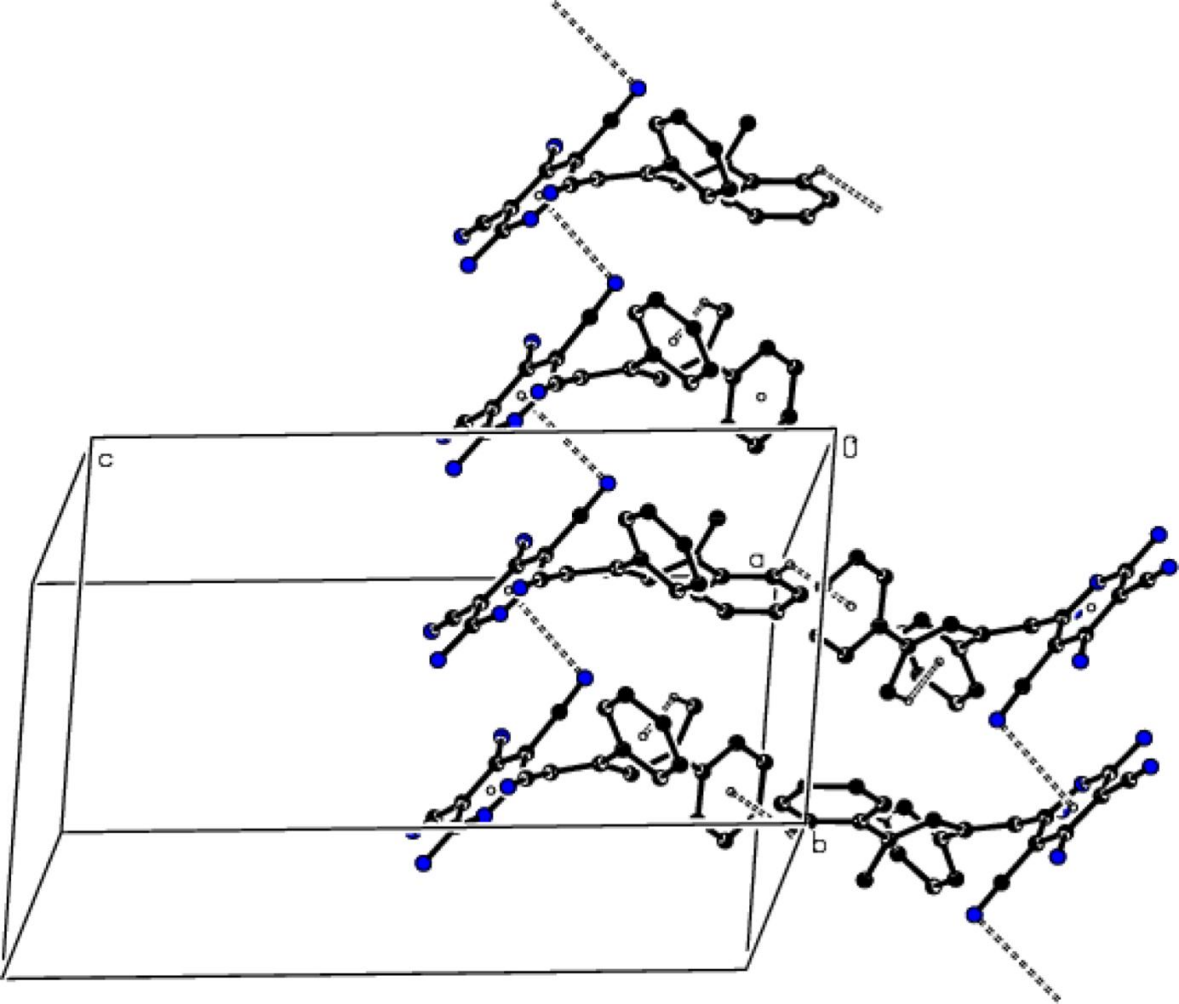
A view of the packing of the title compound along the *a* axis with C—H⋯π inter­actions shown as dashed lines.

In the crystal, the mol­ecules are connected by N—H⋯N and C—H⋯N and O—H⋯N and N—H⋯O hydrogen bonds with each other directly and through water mol­ecules, forming layers parallel to the (001) plane (Table 1 ▸; Figs. 4 ▸, 5 ▸ and 6 ▸). In addition, C—H⋯π inter­actions between these layers ensure the cohesion of the crystal structure (Table 1 ▸; Fig. 7 ▸).

**Figure 8:**
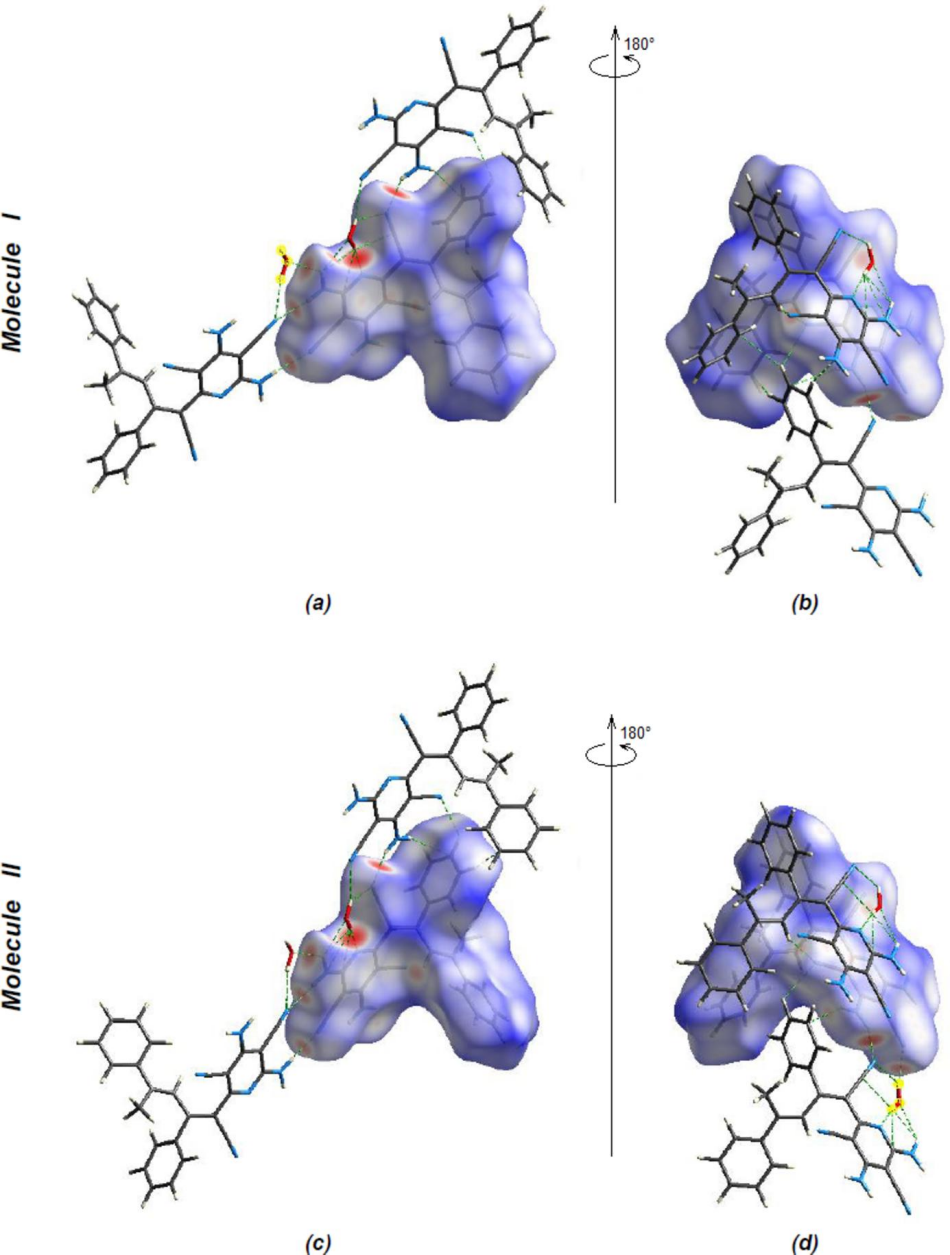
(*a*) Front and (*b*) back sides of the three-dimensional Hirshfeld surface of the title compound mapped over *d_norm_* for **I**, (*c*) front and (*d*) back sides for **II**.

**Figure 9:**
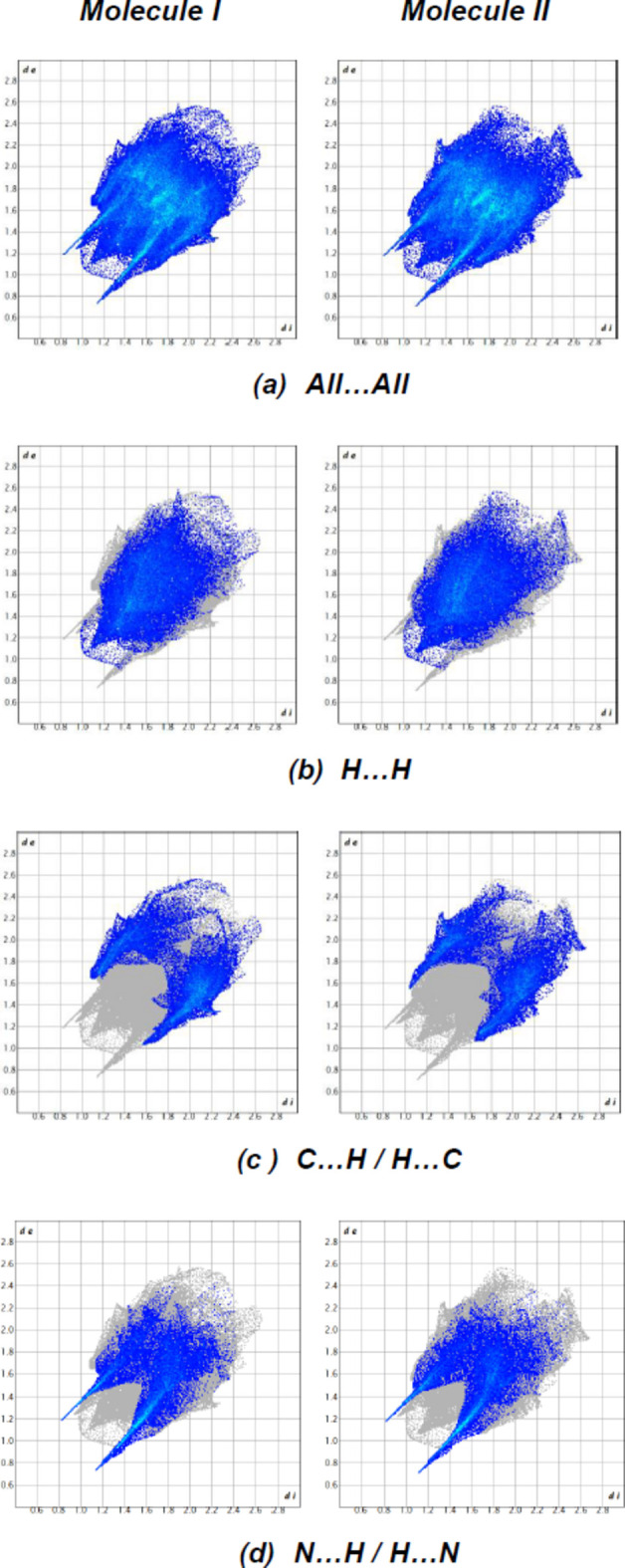
The two-dimensional fingerprint plots, showing (*a*) all inter­actions, and delineated into (*b*) H⋯H, (*c*) C⋯H/H⋯C and (*d*) N⋯H/H⋯N inter­actions. [*d*_e_ and *d*_i_ represent the distances from a point on the Hirshfeld surface to the nearest atoms outside (external) and inside (inter­nal) the surface, respectively.]

*Crystal Explorer 17.5* (Spackman *et al.*, 2021 ▸) was used to generate Hirshfeld surfaces for both independent mol­ecules. The *d*_norm_ mappings for mol­ecules **I** and **II** were performed in the ranges −0.5788 to 1.4167 a.u. and −0.621 to 1.3731 a.u., respectively. The O—H⋯N, N—H⋯O, N—H⋯N and C—H⋯N inter­actions are indicated by red areas on the Hirshfeld surfaces (Fig. 8 ▸ *a*,*b* for **I** and Fig. 8 ▸ *c*,*d* for **II**). Although H⋯H inter­actions (39.1% for mol­ecule **I** and 40.0% for mol­ecule **II**) contribute the most to surface contacts, fingerprint plots (Fig. 9 ▸) show that C⋯H/H⋯C inter­actions (26.6% for mol­ecule **I** and 25.8% for mol­ecule **II**) and N⋯H/H⋯N inter­actions (24.3% for mol­ecules **I** and **II**) are also significant (Tables 1 ▸ and 2 ▸). Other, less notable contacts are C⋯N/N⋯C (4.6% for mol­ecule **I** and 4.4% for mol­ecule **II**), N⋯N (1.9% contribution for mol­ecule **I** and 2.0% for mol­ecule **II**), O⋯H/H⋯O inter­actions (1.6% for mol­ecule **I** and 1.7% for mol­ecule **II**), O⋯C/C⋯O inter­actions (1.0% for mol­ecules **I** and **II**), C⋯C (0.7% for mol­ecule **I** and 0.8% for mol­ecule **II**) and O⋯N/N⋯O inter­actions (0.1% for mol­ecules **I** and **II**). A comparison of the supplied data shows that mol­ecules **I** and **II** have extremely comparable environments.

## 4. Database survey

A search of the Cambridge Structural Database (CSD, version 5.43, update June 2022; Groom *et al.*, 2016 ▸) for the *buta-1,3-diene* unit gave ten similar structures, *viz.* CSD refcode SESRUE (Ibis & Deniz, 2006 ▸), JEYVAL (Ibis & Deniz, 2007*a* ▸), SINDOJ (Ibis & Deniz, 2007*b* ▸), WIHFAV (Ibis & Deniz, 2007*c* ▸), CICMIL (Sathiyanarayanan *et al.*, 2007 ▸), GISDOC (Sathiyanarayanan *et al.*, 2008*a* ▸), GIRQEE (Sathiyanarayanan *et al.*, 2008*b* ▸), IGANUA (Bats *et al.*, 2008 ▸), KABKAB (Narayan *et al.*, 2010 ▸) and IDOTOM (Okuno & Iwahashi, 2013 ▸).

In SESRUE, the butadiene has a conformation closer to cisoid than to transoid, the C4—C3—C2—C1 torsion angle being −64.3 (3)°. In JEYVAL, the butadiene unit has assumed a configuration close to cisoid, but it is not completely planar. The C18—C19— C20—C21 torsion angle is −56.0 (11)°. In SINDOJ, the butadiene unit is not completely planar. The torsional angle of the butadiene unit (C1—C2—C3—C4) is −82.2 (5)°. In WIHFAV, the butadiene unit has assumed a configuration close to cisoid, but is not completely planar. The C4—C3—C2—C1 torsion angle is −97.2 (3)°. In CICMIL, co-operative C—H⋯π inter­actions form mol­ecular dimers. The dimers associate in a one-dimensional chain along the *a-*axis direction. In GISDOC, the torsion angles describing the mol­ecular conformation namely, C2—C1—O1—C7, C8—C7—O1—C1 and O1—C7—C8—C8^i^ [symmetry code: (i) 1 − *x*, 1 − *y*, −*z*] are *trans*, *gauche* and *trans*, respectively. The structure is consolidated by a short intra­molecular C—H⋯O contact. The mol­ecules are held together by C—H⋯π inter­actions, forming a sheet structure parallel to the (201) plane. The structure of GIRQEE is consolidated by a short inter­molecular C—H⋯O contact. Cooperative C—H⋯π inter­actions generate an infinite one-dimensional chains of mol­ecules along the *a*-axis direction. In IGANUA, the asymmetric unit contains two half-mol­ecules. Both complete mol­ecules are generated by crystallographic inversion centres located at the mid-points of the central C—C single bonds; the butadiene groups are planar, with a *trans* conformation about the central C—C bond. The mol­ecules show short intra­molecular H⋯I contacts of 2.89 and 2.92 Å. The crystal packing shows no short inter­molecular contacts. In KABKAB, there are four mol­ecules per unit cell. The symmetrical mol­ecules are arranged in a herringbone fashion (Koren *et al.*, 2003 ▸) in which the mol­ecules are packed in an edge-to-face orientation. In IDOTOM, the mol­ecules are aligned along the *b*-axis. Four kinds of weak C—H⋯N inter­actions are recognized, one of which connects the mol­ecules into a one-dimensional array and the remaining three link these arrays.

## 5. Synthesis and crystallization

A solution of aceto­phenone (17 mmol) and malono­nitrile (26 mmol) in ethanol (35 mL) was stirred for 1 h. Then 5 drops of methyl­piperazine were added to the reaction mixture. The resulting reaction mixture was stirred for 4 h. After the reaction was complete, it was kept for 5 days until the formation of crystals occurred. The crystals were separated by filtration and recrystallized from an ethanol–water solution (m.p. = 458–459 K, yield 55%).

^1^H NMR (300 MHz, DMSO-*d*_6_, ppm.): 2.32 (*s*, 3H, CH_3_); 6.88 (*s*, 4H, 2NH_2_); 7.19–7.87 (*m*, 10H, 10CH_arom._); 7.96 (*s*, 1H, NH). ^13^C NMR (75 MHz, DMSO-*d*_6_, ppm): 17.95 (CH_3_), 61.61 (C_quat._), 67.24 (C_quat._), 69.98 (C_quat._), 116.21 (CN), 116.88 (CN), 117.76 (=CH), 119.32 (CN), 127.52 (2CH_arom._), 127.58 (CH_arom._), 128.62 (2CH_arom._), 128.90 (CH_arom._), 129.37 (2CH_arom._), 129.54 (2CH_arom._), 138.14 (C_arom._), 142.25 (C_arom._), 145.96 (C_quat._), 155.03 (C_quat._), 161.99 (C_quat._), 166.07 (C_quat._), 166.39 (C_quat._).

## 6. Refinement

Crystal data, data collection and structure refinement details are summarized in Table 3 ▸. All C-bound H atoms were placed at calculated positions and refined using a riding model, with C—H = 0.95–0.98 Å, and with *U*_iso_(H) = 1.2 or 1.5*U*_eq_(C). The N-bound H atoms were located in difference-Fourier maps and refined freely. The O-bound H atoms were located in difference-Fourier maps and were refined with *U*_iso_(H) = 1.5*U*_eq_(O). The O—H bond lengths of water mol­ecules were forced to be 0.85 ± 0.02 Å with the DFIX command. Both H atoms of the water mol­ecules were forced to have the same displacement parameters with the EADP command.

## Supplementary Material

Crystal structure: contains datablock(s) I. DOI: 10.1107/S2056989024002962/ox2003sup1.cif

Structure factors: contains datablock(s) I. DOI: 10.1107/S2056989024002962/ox2003Isup2.hkl

Supporting information file. DOI: 10.1107/S2056989024002962/ox2003Isup3.cml

CCDC reference: 2347583

Additional supporting information:  crystallographic information; 3D view; checkCIF report

## Figures and Tables

**Figure 5:**
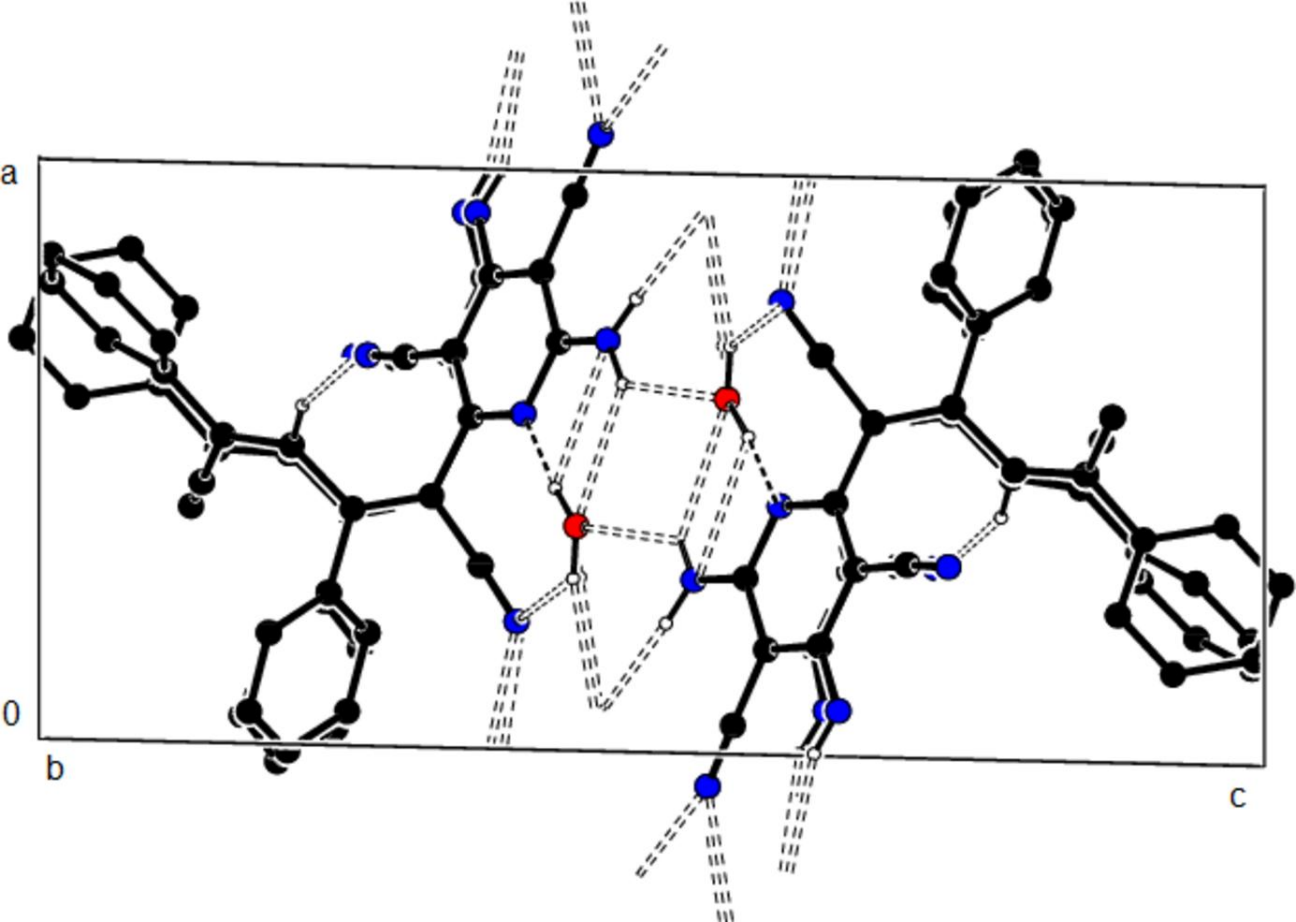
The packing of the title compound viewed along the *b* axis with O—H⋯N, N—H⋯O, N—H⋯N and C—H⋯N hydrogen bonds shown as dashed lines.

**Figure 6:**
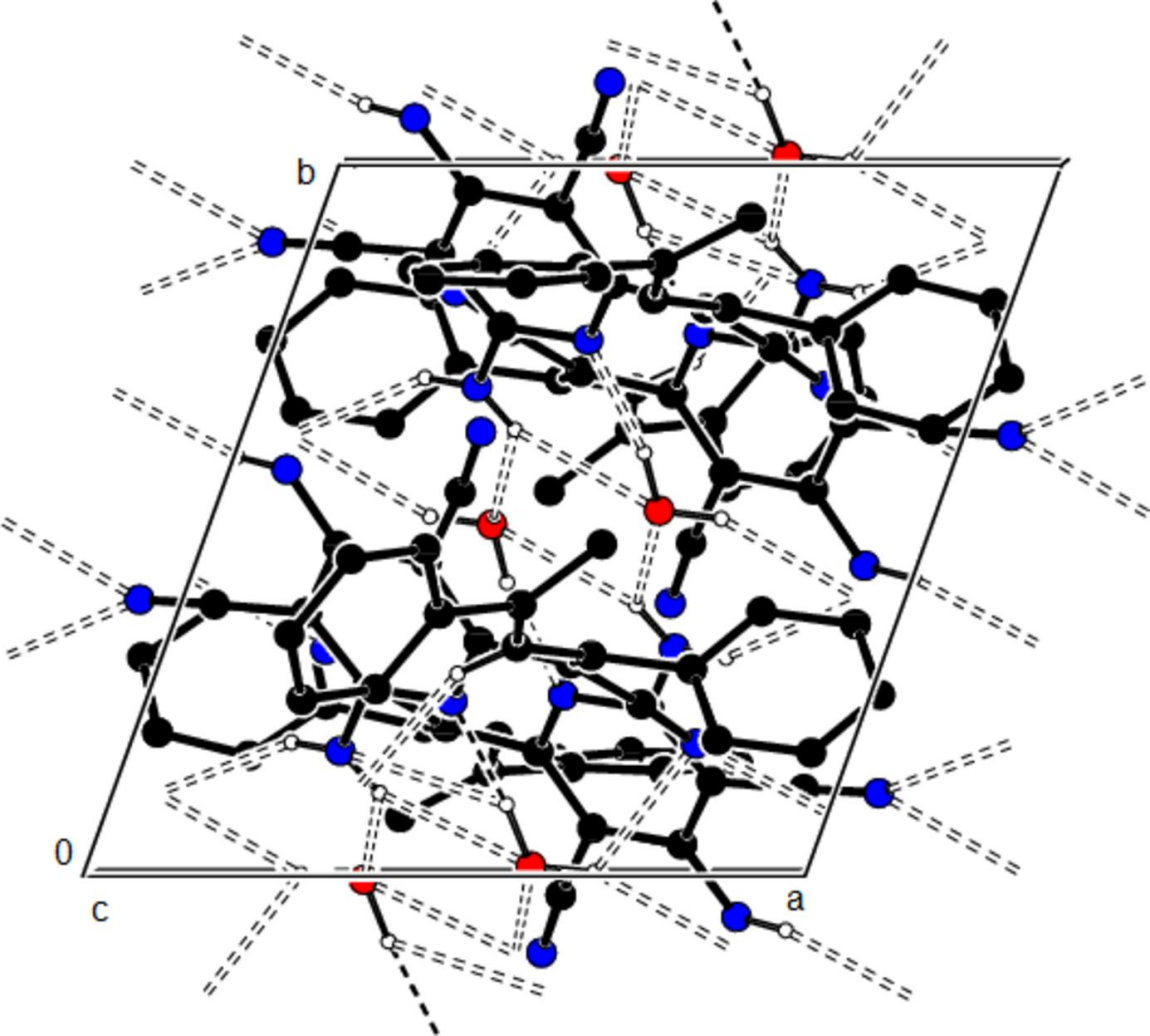
The packing of the title compound viewed along the *c* axis with O—H⋯N, N—H⋯O, N—H⋯N and C—H⋯N hydrogen bonds shown as dashed lines.

**Table 1:** Hydrogen-bond geometry (Å, °) *Cg*5 and *Cg*6 are the centroids of the C14*A*–C19*A* and C20*A*–C25*A* phenyl rings of mol­ecule **II**, respectively.

*D*—H⋯*A*	*D*—H	H⋯*A*	*D*⋯*A*	*D*—H⋯*A*
O1—H1*A*⋯N1	0.93 (2)	1.93 (2)	2.853 (2)	169 (3)
O1—H1*B*⋯N7*A* ^i^	0.89 (2)	2.33 (2)	3.163 (3)	156 (3)
O1*A*—H1*C*⋯N1*A*	1.04 (2)	1.78 (2)	2.811 (3)	174 (2)
O1*A*—H1*D*⋯N7^ii^	0.91 (2)	2.38 (2)	3.206 (3)	152 (2)
O1*A*—H1*D*⋯N13*A*	0.91 (2)	2.59 (2)	3.153 (3)	121 (2)
N2—H2*A*⋯O1	0.86 (3)	2.44 (3)	3.140 (3)	139 (2)
N2—H2*A*⋯O1^iii^	0.86 (3)	2.29 (3)	2.892 (3)	127 (2)
N2—H2*B*⋯N7*A* ^iv^	0.87 (3)	2.41 (3)	3.209 (3)	151.7 (18)
N2*A*—H2*C*⋯O1*A*	0.87 (3)	2.48 (3)	3.174 (3)	137 (2)
N2*A*—H2*C*⋯O1*A* ^v^	0.87 (3)	2.25 (3)	2.859 (3)	127 (3)
N2*A*—H2*D*⋯N7^iv^	0.85 (3)	2.42 (3)	3.205 (3)	154 (3)
N4—H4*A*⋯N13*A* ^vi^	0.82 (3)	2.21 (3)	2.984 (3)	158 (3)
N4*A*—H4*C*⋯N13^vii^	0.84 (3)	2.16 (3)	2.930 (3)	152 (2)
C11—H11⋯N8*A*	0.95	2.59	3.453 (3)	151
C11*A*—H11*A*⋯N8^viii^	0.95	2.49	3.369 (3)	154
C21—H21⋯*Cg*6^ix^	0.95	2.91	3.653 (2)	136
C26*A*—H26*F*⋯*Cg*5	0.98	2.97	3.781 (2)	141

Symmetry codes: (i) 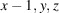 ; (ii) 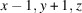 ; (iii) 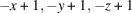 ; (iv) 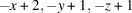 ; (v) 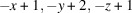 ; (vi) 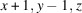 ; (vii) 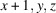 ; (viii) 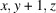 ; (ix) 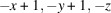 .

**Table 2:** Inter­atomic contacts of the title compound (Å)

N1⋯H1*A*	1.93	*x*, *y*, *z*
H19⋯H26*F*	2.48	*x*, *y*, *z*
H2*A*⋯O1	2.29	1 − *x*, 1 − *y*, 1 − *z*
H2*B*⋯N7*A*	2.41	2 − *x*, 1 − *y*, 1 − *z*
N13⋯H2*D*	2.67	1 − *x*, 1 − *y*, 1 − *z*
H4*A*⋯N13*A*	2.21	1 + *x*, −1 + *y*, *z*
N4⋯H16	2.90	1 + *x*, *y*, *z*
N7⋯H1*D*	2.38	1 + *x*, −1 + *y*, *z*
C7⋯N7	3.21	2 − *x*, −*y*, 1 − *z*
H26*B*⋯H25*A*	2.43	*x*, −1 + *y*, *z*
N13⋯H4*C*	2.16	−1 + *x*, *y*, *z*
C13⋯O1*A*	3.01	*x*, −1 + *y*, *z*
H22⋯H19*A*	2.37	1 − *x*, 1 − *y*, −*z*
N1*A*⋯H1*C*	1.78	*x*, *y*, *z*
H2*C*⋯O1*A*	2.25	1 − *x*, 2 − *y*, 1 − *z*
N4*A*⋯H16*A*	2.69	1 + *x*, *y*, *z*
N7*A*⋯H1*B*	2.33	1 + *x*, *y*, *z*
C7*A*⋯N7*A*	3.21	2 − *x*, 1 − *y*, 1 − *z*
C13*A*⋯O1	3.06	*x*, *y*, *z*

**Table 3:** Experimental details

Crystal data	
Chemical formula	C_25_H_18_N_6_·H_2_O
*M*_r_	420.47
Crystal system, space group	Triclinic, *P* 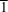
Temperature (K)	100
*a*, *b*, *c* (Å)	10.2188 (5), 10.7365 (5), 20.4119 (10)
α, β, γ (°)	84.376 (2), 89.298 (2), 70.167 (2)
*V* (Å^3^)	2095.97 (18)
*Z*	4
Radiation type	Mo *K*α
μ (mm^−1^)	0.09
Crystal size (mm)	0.32 × 0.19 × 0.16

Data collection	
Diffractometer	Bruker D8 QUEST PHOTON-III CCD
Absorption correction	Multi-scan (*SADABS*; Krause *et al.*, 2015 ▸)
*T*_min_, *T*_max_	0.834, 0.947
No. of measured, independent and observed [*I* > 2σ(*I*)] reflections	55460, 7385, 5414
*R*_int_	0.066
(sin θ/λ)_max_ (Å^−1^)	0.595

Refinement	
*R*[*F* ^2^ > 2σ(*F* ^2^)], *wR*(*F* ^2^), *S*	0.049, 0.143, 1.02
No. of reflections	7385
No. of parameters	625
No. of restraints	4
H-atom treatment	H atoms treated by a mixture of independent and constrained refinement
Δρ_max_, Δρ_min_ (e Å^−3^)	0.39, −0.32

Computer programs: *APEX3* (Bruker, 2018 ▸), *SAINT* (Bruker, 2013 ▸), *SHELXT2014/5* (Sheldrick, 2015*a* ▸), *SHELXL2019/3* (Sheldrick, 2015*b* ▸), *ORTEP-3 for Windows* (Farrugia, 2012 ▸) and *PLATON* (Spek, 2020 ▸).

## supplementary crystallographic information

### Crystal data

**Table:**

C_25_H_18_N_6_·H_2_O	*Z* = 4
*M**_r_* = 420.47	*F*(000) = 880
Triclinic, *P*1	*D*_x_ = 1.332 Mg m^−^^3^
*a* = 10.2188 (5) Å	Mo *K*α radiation, λ = 0.71073 Å
*b* = 10.7365 (5) Å	Cell parameters from 9984 reflections
*c* = 20.4119 (10) Å	θ = 2.3–33.2°
α = 84.376 (2)°	µ = 0.09 mm^−^^1^
β = 89.298 (2)°	*T* = 100 K
γ = 70.167 (2)°	Prism, yellow
*V* = 2095.97 (18) Å^3^	0.32 × 0.19 × 0.16 mm

### Data collection

**Table:**

Bruker D8 QUEST PHOTON-III CCD diffractometer	5414 reflections with *I* > 2σ(*I*)
Radiation source: normal-focus sealed X-ray tube	*R*_int_ = 0.066
φ and ω scans	θ_max_ = 25.0°, θ_min_ = 2.1°
Absorption correction: multi-scan (SADABS; Krause *et al.*, 2015)	*h* = −12→12
*T*_min_ = 0.834, *T*_max_ = 0.947	*k* = −12→12
55460 measured reflections	*l* = −24→24
7385 independent reflections	

### Refinement

**Table:**

Refinement on *F*^2^	4 restraints
Least-squares matrix: full	Hydrogen site location: mixed
*R*[*F*^2^ > 2σ(*F*^2^)] = 0.049	H atoms treated by a mixture of independent and constrained refinement
*wR*(*F*^2^) = 0.143	*w* = 1/[σ^2^(*F*_o_^2^) + (0.0655*P*)^2^ + 1.3358*P*] where *P* = (*F*_o_^2^ + 2*F*_c_^2^)/3
*S* = 1.02	(Δ/σ)_max_ < 0.001
7385 reflections	Δρ_max_ = 0.39 e Å^−^^3^
625 parameters	Δρ_min_ = −0.32 e Å^−^^3^

### Special details

**Table:**

Geometry. All esds (except the esd in the dihedral angle between two l.s. planes) are estimated using the full covariance matrix. The cell esds are taken into account individually in the estimation of esds in distances, angles and torsion angles; correlations between esds in cell parameters are only used when they are defined by crystal symmetry. An approximate (isotropic) treatment of cell esds is used for estimating esds involving l.s. planes.

### Fractional atomic coordinates and isotropic or equivalent isotropic displacement parameters (Å^2^)

**Table:**

	*x*	*y*	*z*	*U*_iso_*/*U*_eq_	
C2	0.6967 (2)	0.2304 (2)	0.42352 (10)	0.0182 (4)	
C3	0.8187 (2)	0.1282 (2)	0.40672 (10)	0.0170 (4)	
C4	0.8094 (2)	0.0389 (2)	0.36291 (10)	0.0171 (4)	
C5	0.67687 (19)	0.0614 (2)	0.33360 (9)	0.0163 (4)	
C6	0.5636 (2)	0.1696 (2)	0.35016 (9)	0.0163 (4)	
C7	0.9508 (2)	0.1177 (2)	0.43378 (10)	0.0193 (4)	
C8	0.6664 (2)	−0.0333 (2)	0.29128 (10)	0.0201 (5)	
C9	0.4236 (2)	0.2037 (2)	0.31930 (10)	0.0170 (4)	
C10	0.3935 (2)	0.20248 (19)	0.25418 (10)	0.0176 (4)	
C11	0.4995 (2)	0.1889 (2)	0.20405 (10)	0.0183 (4)	
H11	0.573998	0.218746	0.213805	0.022*	
C12	0.5041 (2)	0.1392 (2)	0.14563 (10)	0.0196 (4)	
C13	0.3098 (2)	0.2596 (2)	0.36181 (10)	0.0178 (4)	
C14	0.2455 (2)	0.2344 (2)	0.23301 (10)	0.0186 (4)	
C15	0.1649 (2)	0.1629 (2)	0.26180 (10)	0.0236 (5)	
H15	0.204426	0.091894	0.295093	0.028*	
C16	0.0266 (2)	0.1953 (2)	0.24190 (11)	0.0282 (5)	
H16	−0.027569	0.145233	0.260960	0.034*	
C17	−0.0323 (2)	0.3008 (2)	0.19420 (11)	0.0279 (5)	
H17	−0.127240	0.323767	0.181310	0.034*	
C18	0.0467 (2)	0.3726 (2)	0.16541 (11)	0.0261 (5)	
H18	0.006127	0.444966	0.132941	0.031*	
C19	0.1857 (2)	0.3382 (2)	0.18422 (10)	0.0213 (5)	
H19	0.240623	0.385980	0.163584	0.026*	
C20	0.6150 (2)	0.1445 (2)	0.09859 (10)	0.0208 (5)	
C21	0.5886 (2)	0.1566 (2)	0.03092 (11)	0.0313 (5)	
H21	0.500516	0.158680	0.015358	0.038*	
C22	0.6883 (3)	0.1654 (3)	−0.01376 (12)	0.0357 (6)	
H22	0.667793	0.174671	−0.059626	0.043*	
C23	0.8182 (2)	0.1610 (2)	0.00783 (12)	0.0323 (6)	
H23	0.887045	0.166808	−0.022862	0.039*	
C24	0.8457 (2)	0.1479 (3)	0.07461 (12)	0.0340 (6)	
H24	0.934376	0.144405	0.089955	0.041*	
C25	0.7458 (2)	0.1398 (2)	0.11949 (11)	0.0288 (5)	
H25	0.766764	0.130934	0.165288	0.035*	
C26	0.4041 (2)	0.0744 (2)	0.12403 (11)	0.0274 (5)	
H26A	0.360346	0.045193	0.162735	0.041*	
H26B	0.454787	−0.002701	0.100682	0.041*	
H26C	0.332260	0.138534	0.094624	0.041*	
N1	0.57086 (16)	0.24888 (17)	0.39556 (8)	0.0189 (4)	
N2	0.7017 (2)	0.31559 (19)	0.46583 (9)	0.0231 (4)	
H2A	0.628 (3)	0.376 (3)	0.4779 (12)	0.035 (7)*	
H2B	0.778 (3)	0.303 (2)	0.4884 (11)	0.025 (6)*	
N4	0.92089 (19)	−0.06329 (18)	0.34776 (10)	0.0225 (4)	
H4A	0.996 (3)	−0.082 (3)	0.3675 (13)	0.038 (8)*	
H4B	0.917 (2)	−0.119 (3)	0.3188 (12)	0.032 (7)*	
N7	1.05707 (18)	0.11177 (18)	0.45446 (9)	0.0270 (4)	
N8	0.66825 (18)	−0.11456 (18)	0.25793 (9)	0.0259 (4)	
N13	0.21914 (17)	0.31331 (18)	0.39406 (9)	0.0232 (4)	
C2A	0.7035 (2)	0.7391 (2)	0.42403 (10)	0.0185 (4)	
C3A	0.8244 (2)	0.6325 (2)	0.41013 (10)	0.0171 (4)	
C4A	0.8140 (2)	0.5382 (2)	0.36907 (10)	0.0175 (4)	
C5A	0.68280 (19)	0.56133 (19)	0.33876 (9)	0.0161 (4)	
C6A	0.5711 (2)	0.6731 (2)	0.35225 (9)	0.0165 (4)	
C7A	0.9562 (2)	0.6215 (2)	0.43738 (10)	0.0192 (4)	
C8A	0.6711 (2)	0.4623 (2)	0.29911 (11)	0.0233 (5)	
C9A	0.4330 (2)	0.7034 (2)	0.32019 (10)	0.0175 (4)	
C10A	0.4078 (2)	0.6919 (2)	0.25576 (10)	0.0184 (4)	
C11A	0.5150 (2)	0.6787 (2)	0.20631 (10)	0.0195 (4)	
H11A	0.583615	0.716833	0.214947	0.023*	
C12A	0.5295 (2)	0.6195 (2)	0.14987 (10)	0.0207 (5)	
C13A	0.3151 (2)	0.7614 (2)	0.36033 (10)	0.0188 (4)	
C14A	0.2620 (2)	0.7106 (2)	0.23633 (10)	0.0200 (5)	
C15A	0.1942 (2)	0.6297 (2)	0.26811 (11)	0.0256 (5)	
H15A	0.242002	0.561894	0.301507	0.031*	
C16A	0.0578 (2)	0.6474 (3)	0.25135 (12)	0.0337 (6)	
H16A	0.012085	0.592019	0.273149	0.040*	
C17A	−0.0118 (2)	0.7464 (3)	0.20266 (12)	0.0394 (7)	
H17A	−0.105164	0.758347	0.190777	0.047*	
C18A	0.0544 (2)	0.8275 (3)	0.17141 (12)	0.0374 (6)	
H18A	0.005698	0.896110	0.138553	0.045*	
C19A	0.1911 (2)	0.8099 (2)	0.18749 (10)	0.0264 (5)	
H19A	0.236416	0.865256	0.165300	0.032*	
C20A	0.6380 (2)	0.6311 (2)	0.10246 (10)	0.0213 (5)	
C21A	0.6895 (2)	0.5393 (2)	0.05612 (11)	0.0294 (5)	
H21A	0.657262	0.466143	0.055805	0.035*	
C22A	0.7869 (2)	0.5535 (3)	0.01061 (12)	0.0354 (6)	
H22A	0.821174	0.489848	−0.020243	0.042*	
C23A	0.8344 (2)	0.6602 (3)	0.01009 (11)	0.0320 (6)	
H23A	0.900355	0.670274	−0.021299	0.038*	
C24A	0.7851 (2)	0.7518 (3)	0.05550 (11)	0.0318 (6)	
H24A	0.817552	0.824936	0.055411	0.038*	
C25A	0.6884 (2)	0.7373 (2)	0.10118 (11)	0.0268 (5)	
H25A	0.655749	0.800735	0.132239	0.032*	
C26A	0.4477 (2)	0.5341 (2)	0.13285 (11)	0.0282 (5)	
H26D	0.510380	0.441971	0.132207	0.042*	
H26E	0.402977	0.566782	0.089358	0.042*	
H26F	0.376437	0.537615	0.165853	0.042*	
N1A	0.57830 (17)	0.75733 (17)	0.39527 (8)	0.0199 (4)	
N2A	0.7090 (2)	0.82797 (19)	0.46381 (9)	0.0242 (4)	
H2C	0.634 (3)	0.887 (3)	0.4764 (13)	0.039 (8)*	
H2D	0.781 (3)	0.816 (3)	0.4873 (13)	0.036 (7)*	
N4A	0.92293 (19)	0.43097 (19)	0.35784 (10)	0.0233 (4)	
H4C	0.998 (3)	0.413 (2)	0.3793 (12)	0.030 (7)*	
H4D	0.916 (3)	0.375 (3)	0.3307 (13)	0.034 (7)*	
N7A	1.06263 (18)	0.61477 (18)	0.45827 (9)	0.0262 (4)	
N8A	0.67225 (19)	0.37721 (19)	0.26829 (10)	0.0286 (4)	
N13A	0.21990 (18)	0.81696 (18)	0.38964 (9)	0.0248 (4)	
O1	0.38740 (17)	0.48954 (17)	0.43987 (9)	0.0365 (4)	
H1A	0.437 (3)	0.408 (2)	0.4247 (13)	0.050 (6)*	
H1B	0.2974 (19)	0.501 (3)	0.4414 (14)	0.050 (6)*	
O1A	0.38751 (17)	0.98991 (18)	0.43855 (9)	0.0392 (4)	
H1C	0.452 (2)	0.9031 (19)	0.4212 (11)	0.035 (5)*	
H1D	0.2966 (18)	0.998 (2)	0.4359 (12)	0.035 (5)*	

### Atomic displacement parameters (Å^2^)

**Table:**

	*U*^11^	*U*^22^	*U*^33^	*U*^12^	*U*^13^	*U*^23^
C2	0.0181 (10)	0.0202 (11)	0.0138 (10)	−0.0040 (9)	−0.0006 (8)	0.0019 (8)
C3	0.0146 (10)	0.0189 (11)	0.0169 (10)	−0.0051 (8)	−0.0020 (8)	−0.0007 (8)
C4	0.0165 (10)	0.0180 (11)	0.0158 (10)	−0.0051 (8)	0.0003 (8)	0.0006 (8)
C5	0.0132 (10)	0.0194 (11)	0.0157 (10)	−0.0044 (8)	−0.0016 (8)	−0.0022 (8)
C6	0.0143 (10)	0.0192 (11)	0.0149 (10)	−0.0052 (8)	−0.0006 (8)	0.0003 (8)
C7	0.0181 (11)	0.0192 (11)	0.0195 (11)	−0.0052 (9)	−0.0011 (9)	−0.0001 (9)
C8	0.0144 (10)	0.0213 (11)	0.0229 (11)	−0.0041 (9)	−0.0017 (9)	0.0003 (9)
C9	0.0147 (10)	0.0174 (10)	0.0188 (10)	−0.0051 (8)	0.0004 (8)	−0.0024 (8)
C10	0.0167 (10)	0.0164 (10)	0.0198 (11)	−0.0060 (8)	−0.0010 (8)	−0.0016 (8)
C11	0.0141 (10)	0.0207 (11)	0.0195 (11)	−0.0055 (8)	−0.0027 (8)	−0.0006 (8)
C12	0.0177 (10)	0.0189 (11)	0.0191 (11)	−0.0026 (9)	−0.0035 (8)	−0.0001 (8)
C13	0.0149 (10)	0.0193 (11)	0.0183 (11)	−0.0049 (9)	−0.0046 (9)	0.0005 (9)
C14	0.0155 (10)	0.0219 (11)	0.0180 (10)	−0.0046 (9)	−0.0003 (8)	−0.0066 (9)
C15	0.0234 (11)	0.0296 (12)	0.0204 (11)	−0.0118 (10)	−0.0003 (9)	−0.0042 (9)
C16	0.0228 (11)	0.0419 (14)	0.0268 (12)	−0.0183 (11)	0.0048 (10)	−0.0104 (11)
C17	0.0164 (11)	0.0428 (14)	0.0248 (12)	−0.0077 (10)	−0.0020 (9)	−0.0122 (10)
C18	0.0206 (11)	0.0323 (13)	0.0212 (11)	−0.0023 (10)	−0.0041 (9)	−0.0074 (10)
C19	0.0186 (10)	0.0259 (12)	0.0195 (11)	−0.0070 (9)	0.0000 (9)	−0.0043 (9)
C20	0.0216 (11)	0.0185 (11)	0.0195 (11)	−0.0032 (9)	−0.0010 (9)	−0.0021 (8)
C21	0.0264 (12)	0.0387 (14)	0.0255 (12)	−0.0064 (11)	−0.0028 (10)	−0.0051 (10)
C22	0.0396 (14)	0.0441 (15)	0.0203 (12)	−0.0107 (12)	0.0013 (11)	−0.0019 (11)
C23	0.0326 (13)	0.0373 (14)	0.0273 (13)	−0.0123 (11)	0.0099 (10)	−0.0052 (11)
C24	0.0280 (13)	0.0455 (15)	0.0317 (13)	−0.0157 (12)	0.0042 (11)	−0.0083 (11)
C25	0.0251 (12)	0.0395 (14)	0.0228 (12)	−0.0114 (11)	−0.0002 (10)	−0.0063 (10)
C26	0.0254 (12)	0.0298 (13)	0.0293 (13)	−0.0100 (10)	0.0010 (10)	−0.0111 (10)
N1	0.0138 (8)	0.0222 (9)	0.0181 (9)	−0.0024 (7)	−0.0013 (7)	−0.0029 (7)
N2	0.0184 (10)	0.0271 (11)	0.0207 (10)	−0.0020 (9)	−0.0028 (8)	−0.0085 (8)
N4	0.0142 (9)	0.0222 (10)	0.0290 (11)	−0.0016 (8)	−0.0005 (8)	−0.0086 (8)
N7	0.0211 (10)	0.0275 (11)	0.0325 (11)	−0.0092 (8)	−0.0067 (8)	0.0006 (8)
N8	0.0240 (10)	0.0246 (10)	0.0298 (11)	−0.0082 (8)	−0.0013 (8)	−0.0058 (9)
N13	0.0150 (9)	0.0290 (10)	0.0224 (10)	−0.0030 (8)	−0.0019 (8)	−0.0029 (8)
C2A	0.0187 (10)	0.0206 (11)	0.0137 (10)	−0.0039 (9)	0.0004 (8)	0.0011 (8)
C3A	0.0139 (10)	0.0185 (11)	0.0170 (10)	−0.0036 (8)	−0.0025 (8)	0.0008 (8)
C4A	0.0152 (10)	0.0202 (11)	0.0152 (10)	−0.0045 (8)	0.0004 (8)	0.0012 (8)
C5A	0.0130 (10)	0.0185 (11)	0.0159 (10)	−0.0045 (8)	−0.0004 (8)	−0.0009 (8)
C6A	0.0150 (10)	0.0201 (11)	0.0129 (10)	−0.0047 (8)	0.0002 (8)	0.0011 (8)
C7A	0.0196 (11)	0.0172 (11)	0.0194 (11)	−0.0050 (9)	−0.0003 (9)	−0.0005 (8)
C8A	0.0161 (10)	0.0252 (12)	0.0273 (12)	−0.0059 (9)	−0.0001 (9)	−0.0009 (10)
C9A	0.0138 (10)	0.0190 (11)	0.0186 (10)	−0.0044 (8)	0.0007 (8)	−0.0008 (8)
C10A	0.0163 (10)	0.0176 (11)	0.0206 (11)	−0.0049 (8)	−0.0016 (8)	−0.0015 (8)
C11A	0.0149 (10)	0.0257 (12)	0.0194 (11)	−0.0086 (9)	−0.0008 (8)	−0.0028 (9)
C12A	0.0172 (10)	0.0234 (11)	0.0203 (11)	−0.0050 (9)	−0.0021 (9)	−0.0019 (9)
C13A	0.0150 (10)	0.0221 (11)	0.0178 (11)	−0.0047 (9)	−0.0052 (9)	−0.0006 (9)
C14A	0.0164 (10)	0.0274 (12)	0.0169 (10)	−0.0073 (9)	−0.0009 (8)	−0.0064 (9)
C15A	0.0244 (11)	0.0318 (13)	0.0248 (12)	−0.0139 (10)	0.0035 (9)	−0.0068 (10)
C16A	0.0285 (13)	0.0541 (17)	0.0305 (13)	−0.0257 (12)	0.0101 (11)	−0.0197 (12)
C17A	0.0174 (12)	0.073 (2)	0.0317 (14)	−0.0149 (13)	0.0028 (10)	−0.0273 (14)
C18A	0.0212 (12)	0.0588 (17)	0.0228 (12)	−0.0003 (12)	−0.0054 (10)	−0.0085 (12)
C19A	0.0228 (11)	0.0351 (13)	0.0189 (11)	−0.0063 (10)	−0.0007 (9)	−0.0047 (10)
C20A	0.0150 (10)	0.0285 (12)	0.0189 (11)	−0.0049 (9)	−0.0033 (8)	−0.0031 (9)
C21A	0.0265 (12)	0.0346 (14)	0.0244 (12)	−0.0058 (10)	−0.0010 (10)	−0.0075 (10)
C22A	0.0287 (13)	0.0467 (16)	0.0244 (13)	−0.0027 (11)	0.0024 (10)	−0.0112 (11)
C23A	0.0197 (11)	0.0522 (16)	0.0221 (12)	−0.0096 (11)	0.0035 (9)	−0.0032 (11)
C24A	0.0247 (12)	0.0484 (15)	0.0237 (12)	−0.0157 (11)	−0.0029 (10)	0.0016 (11)
C25A	0.0206 (11)	0.0387 (14)	0.0209 (11)	−0.0094 (10)	−0.0018 (9)	−0.0036 (10)
C26A	0.0307 (12)	0.0297 (13)	0.0296 (13)	−0.0150 (10)	0.0051 (10)	−0.0113 (10)
N1A	0.0153 (8)	0.0246 (10)	0.0169 (9)	−0.0025 (7)	−0.0002 (7)	−0.0034 (7)
N2A	0.0220 (10)	0.0257 (11)	0.0203 (10)	−0.0004 (9)	−0.0026 (9)	−0.0078 (8)
N4A	0.0146 (9)	0.0227 (10)	0.0299 (11)	−0.0015 (8)	−0.0027 (8)	−0.0081 (9)
N7A	0.0193 (10)	0.0283 (11)	0.0305 (11)	−0.0080 (8)	−0.0061 (8)	−0.0003 (8)
N8A	0.0254 (10)	0.0273 (11)	0.0335 (11)	−0.0094 (9)	−0.0015 (8)	−0.0041 (9)
N13A	0.0144 (9)	0.0303 (11)	0.0257 (10)	−0.0021 (8)	−0.0011 (8)	−0.0044 (8)
O1	0.0206 (9)	0.0327 (10)	0.0548 (12)	−0.0034 (8)	−0.0032 (8)	−0.0187 (9)
O1A	0.0239 (9)	0.0356 (10)	0.0575 (12)	−0.0055 (8)	−0.0034 (8)	−0.0185 (9)

### Geometric parameters (Å, º)

**Table:**

C2—N2	1.331 (3)	C2A—C3A	1.419 (3)
C2—N1	1.357 (3)	C3A—C4A	1.407 (3)
C2—C3	1.418 (3)	C3A—C7A	1.427 (3)
C3—C4	1.400 (3)	C4A—N4A	1.339 (3)
C3—C7	1.429 (3)	C4A—C5A	1.416 (3)
C4—N4	1.344 (3)	C5A—C6A	1.395 (3)
C4—C5	1.421 (3)	C5A—C8A	1.434 (3)
C5—C6	1.399 (3)	C6A—N1A	1.340 (3)
C5—C8	1.428 (3)	C6A—C9A	1.482 (3)
C6—N1	1.337 (3)	C7A—N7A	1.149 (3)
C6—C9	1.483 (3)	C8A—N8A	1.156 (3)
C7—N7	1.149 (3)	C9A—C10A	1.369 (3)
C8—N8	1.153 (3)	C9A—C13A	1.441 (3)
C9—C10	1.370 (3)	C10A—C11A	1.461 (3)
C9—C13	1.439 (3)	C10A—C14A	1.488 (3)
C10—C11	1.462 (3)	C11A—C12A	1.352 (3)
C10—C14	1.492 (3)	C11A—H11A	0.9500
C11—C12	1.348 (3)	C12A—C20A	1.492 (3)
C11—H11	0.9500	C12A—C26A	1.500 (3)
C12—C20	1.489 (3)	C13A—N13A	1.149 (3)
C12—C26	1.508 (3)	C14A—C19A	1.393 (3)
C13—N13	1.149 (3)	C14A—C15A	1.393 (3)
C14—C15	1.394 (3)	C15A—C16A	1.384 (3)
C14—C19	1.394 (3)	C15A—H15A	0.9500
C15—C16	1.391 (3)	C16A—C17A	1.385 (4)
C15—H15	0.9500	C16A—H16A	0.9500
C16—C17	1.389 (3)	C17A—C18A	1.380 (4)
C16—H16	0.9500	C17A—H17A	0.9500
C17—C18	1.383 (3)	C18A—C19A	1.383 (3)
C17—H17	0.9500	C18A—H18A	0.9500
C18—C19	1.390 (3)	C19A—H19A	0.9500
C18—H18	0.9500	C20A—C25A	1.399 (3)
C19—H19	0.9500	C20A—C21A	1.401 (3)
C20—C25	1.391 (3)	C21A—C22A	1.389 (3)
C20—C21	1.396 (3)	C21A—H21A	0.9500
C21—C22	1.380 (3)	C22A—C23A	1.386 (3)
C21—H21	0.9500	C22A—H22A	0.9500
C22—C23	1.388 (3)	C23A—C24A	1.383 (3)
C22—H22	0.9500	C23A—H23A	0.9500
C23—C24	1.379 (3)	C24A—C25A	1.387 (3)
C23—H23	0.9500	C24A—H24A	0.9500
C24—C25	1.382 (3)	C25A—H25A	0.9500
C24—H24	0.9500	C26A—H26D	0.9800
C25—H25	0.9500	C26A—H26E	0.9800
C26—H26A	0.9800	C26A—H26F	0.9800
C26—H26B	0.9800	N2A—H2C	0.87 (3)
C26—H26C	0.9800	N2A—H2D	0.85 (3)
N2—H2A	0.86 (3)	N4A—H4C	0.84 (3)
N2—H2B	0.88 (2)	N4A—H4D	0.88 (3)
N4—H4A	0.83 (3)	O1—H1A	0.930 (17)
N4—H4B	0.89 (3)	O1—H1B	0.886 (17)
C2A—N2A	1.328 (3)	O1A—H1C	1.035 (16)
C2A—N1A	1.358 (3)	O1A—H1D	0.904 (16)
			
N2—C2—N1	117.04 (18)	N2A—C2A—C3A	121.37 (19)
N2—C2—C3	121.22 (18)	N1A—C2A—C3A	121.39 (18)
N1—C2—C3	121.70 (18)	C4A—C3A—C2A	119.78 (17)
C4—C3—C2	119.73 (17)	C4A—C3A—C7A	120.18 (18)
C4—C3—C7	120.23 (18)	C2A—C3A—C7A	120.03 (18)
C2—C3—C7	120.04 (18)	N4A—C4A—C3A	122.20 (18)
N4—C4—C3	122.02 (18)	N4A—C4A—C5A	120.38 (19)
N4—C4—C5	120.57 (19)	C3A—C4A—C5A	117.41 (18)
C3—C4—C5	117.39 (18)	C6A—C5A—C4A	118.84 (18)
C6—C5—C4	118.87 (17)	C6A—C5A—C8A	123.79 (17)
C6—C5—C8	123.83 (17)	C4A—C5A—C8A	117.23 (18)
C4—C5—C8	117.22 (17)	N1A—C6A—C5A	123.76 (17)
N1—C6—C5	123.49 (17)	N1A—C6A—C9A	115.48 (17)
N1—C6—C9	114.35 (17)	C5A—C6A—C9A	120.74 (17)
C5—C6—C9	122.14 (17)	N7A—C7A—C3A	178.5 (2)
N7—C7—C3	178.3 (2)	N8A—C8A—C5A	174.8 (2)
N8—C8—C5	175.0 (2)	C10A—C9A—C13A	117.87 (17)
C10—C9—C13	117.96 (17)	C10A—C9A—C6A	126.45 (18)
C10—C9—C6	126.87 (18)	C13A—C9A—C6A	115.45 (17)
C13—C9—C6	114.65 (17)	C9A—C10A—C11A	121.54 (18)
C9—C10—C11	121.47 (17)	C9A—C10A—C14A	117.15 (18)
C9—C10—C14	118.91 (18)	C11A—C10A—C14A	120.87 (17)
C11—C10—C14	119.08 (17)	C12A—C11A—C10A	128.63 (19)
C12—C11—C10	127.66 (18)	C12A—C11A—H11A	115.7
C12—C11—H11	116.2	C10A—C11A—H11A	115.7
C10—C11—H11	116.2	C11A—C12A—C20A	119.58 (18)
C11—C12—C20	119.53 (18)	C11A—C12A—C26A	123.85 (19)
C11—C12—C26	124.22 (19)	C20A—C12A—C26A	116.47 (18)
C20—C12—C26	116.24 (18)	N13A—C13A—C9A	174.7 (2)
N13—C13—C9	174.9 (2)	C19A—C14A—C15A	119.38 (19)
C15—C14—C19	119.10 (19)	C19A—C14A—C10A	120.81 (19)
C15—C14—C10	121.07 (19)	C15A—C14A—C10A	119.80 (19)
C19—C14—C10	119.82 (18)	C16A—C15A—C14A	120.4 (2)
C16—C15—C14	120.2 (2)	C16A—C15A—H15A	119.8
C16—C15—H15	119.9	C14A—C15A—H15A	119.8
C14—C15—H15	119.9	C15A—C16A—C17A	119.8 (2)
C17—C16—C15	120.0 (2)	C15A—C16A—H16A	120.1
C17—C16—H16	120.0	C17A—C16A—H16A	120.1
C15—C16—H16	120.0	C18A—C17A—C16A	120.1 (2)
C18—C17—C16	120.3 (2)	C18A—C17A—H17A	120.0
C18—C17—H17	119.9	C16A—C17A—H17A	120.0
C16—C17—H17	119.9	C17A—C18A—C19A	120.6 (2)
C17—C18—C19	119.7 (2)	C17A—C18A—H18A	119.7
C17—C18—H18	120.2	C19A—C18A—H18A	119.7
C19—C18—H18	120.2	C18A—C19A—C14A	119.8 (2)
C18—C19—C14	120.7 (2)	C18A—C19A—H19A	120.1
C18—C19—H19	119.6	C14A—C19A—H19A	120.1
C14—C19—H19	119.6	C25A—C20A—C21A	117.5 (2)
C25—C20—C21	117.8 (2)	C25A—C20A—C12A	120.99 (19)
C25—C20—C12	122.29 (18)	C21A—C20A—C12A	121.5 (2)
C21—C20—C12	119.92 (19)	C22A—C21A—C20A	121.1 (2)
C22—C21—C20	121.1 (2)	C22A—C21A—H21A	119.4
C22—C21—H21	119.5	C20A—C21A—H21A	119.4
C20—C21—H21	119.5	C23A—C22A—C21A	120.2 (2)
C21—C22—C23	120.5 (2)	C23A—C22A—H22A	119.9
C21—C22—H22	119.8	C21A—C22A—H22A	119.9
C23—C22—H22	119.8	C24A—C23A—C22A	119.6 (2)
C24—C23—C22	118.8 (2)	C24A—C23A—H23A	120.2
C24—C23—H23	120.6	C22A—C23A—H23A	120.2
C22—C23—H23	120.6	C23A—C24A—C25A	120.2 (2)
C23—C24—C25	120.8 (2)	C23A—C24A—H24A	119.9
C23—C24—H24	119.6	C25A—C24A—H24A	119.9
C25—C24—H24	119.6	C24A—C25A—C20A	121.4 (2)
C24—C25—C20	121.0 (2)	C24A—C25A—H25A	119.3
C24—C25—H25	119.5	C20A—C25A—H25A	119.3
C20—C25—H25	119.5	C12A—C26A—H26D	109.5
C12—C26—H26A	109.5	C12A—C26A—H26E	109.5
C12—C26—H26B	109.5	H26D—C26A—H26E	109.5
H26A—C26—H26B	109.5	C12A—C26A—H26F	109.5
C12—C26—H26C	109.5	H26D—C26A—H26F	109.5
H26A—C26—H26C	109.5	H26E—C26A—H26F	109.5
H26B—C26—H26C	109.5	C6A—N1A—C2A	118.50 (17)
C6—N1—C2	118.52 (17)	C2A—N2A—H2C	121.7 (17)
C2—N2—H2A	122.3 (17)	C2A—N2A—H2D	121.1 (17)
C2—N2—H2B	120.7 (15)	H2C—N2A—H2D	115 (2)
H2A—N2—H2B	116 (2)	C4A—N4A—H4C	120.0 (17)
C4—N4—H4A	121.1 (18)	C4A—N4A—H4D	121.6 (17)
C4—N4—H4B	122.8 (16)	H4C—N4A—H4D	118 (2)
H4A—N4—H4B	116 (2)	H1A—O1—H1B	111 (2)
N2A—C2A—N1A	117.19 (19)	H1C—O1A—H1D	113 (2)
			
N2—C2—C3—C4	178.88 (19)	N2A—C2A—C3A—C4A	178.38 (19)
N1—C2—C3—C4	−3.6 (3)	N1A—C2A—C3A—C4A	−4.1 (3)
N2—C2—C3—C7	−1.9 (3)	N2A—C2A—C3A—C7A	−2.1 (3)
N1—C2—C3—C7	175.66 (18)	N1A—C2A—C3A—C7A	175.42 (18)
C2—C3—C4—N4	−176.88 (19)	C2A—C3A—C4A—N4A	−175.52 (19)
C7—C3—C4—N4	3.9 (3)	C7A—C3A—C4A—N4A	4.9 (3)
C2—C3—C4—C5	4.3 (3)	C2A—C3A—C4A—C5A	5.2 (3)
C7—C3—C4—C5	−174.93 (18)	C7A—C3A—C4A—C5A	−174.31 (18)
N4—C4—C5—C6	−179.39 (19)	N4A—C4A—C5A—C6A	179.16 (19)
C3—C4—C5—C6	−0.5 (3)	C3A—C4A—C5A—C6A	−1.6 (3)
N4—C4—C5—C8	3.9 (3)	N4A—C4A—C5A—C8A	3.2 (3)
C3—C4—C5—C8	−177.24 (18)	C3A—C4A—C5A—C8A	−177.52 (18)
C4—C5—C6—N1	−4.5 (3)	C4A—C5A—C6A—N1A	−3.7 (3)
C8—C5—C6—N1	172.01 (19)	C8A—C5A—C6A—N1A	171.99 (19)
C4—C5—C6—C9	176.96 (18)	C4A—C5A—C6A—C9A	178.24 (18)
C8—C5—C6—C9	−6.6 (3)	C8A—C5A—C6A—C9A	−6.1 (3)
N1—C6—C9—C10	139.7 (2)	N1A—C6A—C9A—C10A	140.7 (2)
C5—C6—C9—C10	−41.6 (3)	C5A—C6A—C9A—C10A	−41.1 (3)
N1—C6—C9—C13	−31.8 (2)	N1A—C6A—C9A—C13A	−33.6 (3)
C5—C6—C9—C13	146.87 (19)	C5A—C6A—C9A—C13A	144.61 (19)
C13—C9—C10—C11	160.02 (19)	C13A—C9A—C10A—C11A	158.67 (19)
C6—C9—C10—C11	−11.2 (3)	C6A—C9A—C10A—C11A	−15.5 (3)
C13—C9—C10—C14	−11.4 (3)	C13A—C9A—C10A—C14A	−13.7 (3)
C6—C9—C10—C14	177.30 (18)	C6A—C9A—C10A—C14A	172.10 (19)
C9—C10—C11—C12	153.8 (2)	C9A—C10A—C11A—C12A	153.0 (2)
C14—C10—C11—C12	−34.7 (3)	C14A—C10A—C11A—C12A	−34.9 (3)
C10—C11—C12—C20	174.73 (19)	C10A—C11A—C12A—C20A	173.0 (2)
C10—C11—C12—C26	−6.4 (3)	C10A—C11A—C12A—C26A	−10.8 (4)
C9—C10—C14—C15	−56.0 (3)	C9A—C10A—C14A—C19A	120.8 (2)
C11—C10—C14—C15	132.3 (2)	C11A—C10A—C14A—C19A	−51.6 (3)
C9—C10—C14—C19	123.2 (2)	C9A—C10A—C14A—C15A	−57.9 (3)
C11—C10—C14—C19	−48.5 (3)	C11A—C10A—C14A—C15A	129.6 (2)
C19—C14—C15—C16	0.0 (3)	C19A—C14A—C15A—C16A	0.1 (3)
C10—C14—C15—C16	179.21 (19)	C10A—C14A—C15A—C16A	178.87 (19)
C14—C15—C16—C17	−1.3 (3)	C14A—C15A—C16A—C17A	0.0 (3)
C15—C16—C17—C18	1.2 (3)	C15A—C16A—C17A—C18A	−0.5 (4)
C16—C17—C18—C19	0.3 (3)	C16A—C17A—C18A—C19A	1.0 (4)
C17—C18—C19—C14	−1.7 (3)	C17A—C18A—C19A—C14A	−0.9 (3)
C15—C14—C19—C18	1.5 (3)	C15A—C14A—C19A—C18A	0.4 (3)
C10—C14—C19—C18	−177.72 (19)	C10A—C14A—C19A—C18A	−178.4 (2)
C11—C12—C20—C25	30.2 (3)	C11A—C12A—C20A—C25A	−24.1 (3)
C26—C12—C20—C25	−148.8 (2)	C26A—C12A—C20A—C25A	159.4 (2)
C11—C12—C20—C21	−148.9 (2)	C11A—C12A—C20A—C21A	158.2 (2)
C26—C12—C20—C21	32.2 (3)	C26A—C12A—C20A—C21A	−18.3 (3)
C25—C20—C21—C22	−0.9 (3)	C25A—C20A—C21A—C22A	−0.1 (3)
C12—C20—C21—C22	178.2 (2)	C12A—C20A—C21A—C22A	177.7 (2)
C20—C21—C22—C23	0.8 (4)	C20A—C21A—C22A—C23A	−0.4 (3)
C21—C22—C23—C24	−0.2 (4)	C21A—C22A—C23A—C24A	0.6 (3)
C22—C23—C24—C25	−0.2 (4)	C22A—C23A—C24A—C25A	−0.2 (3)
C23—C24—C25—C20	0.1 (4)	C23A—C24A—C25A—C20A	−0.3 (3)
C21—C20—C25—C24	0.5 (3)	C21A—C20A—C25A—C24A	0.4 (3)
C12—C20—C25—C24	−178.6 (2)	C12A—C20A—C25A—C24A	−177.3 (2)
C5—C6—N1—C2	5.3 (3)	C5A—C6A—N1A—C2A	5.0 (3)
C9—C6—N1—C2	−175.98 (17)	C9A—C6A—N1A—C2A	−176.85 (17)
N2—C2—N1—C6	176.39 (18)	N2A—C2A—N1A—C6A	176.61 (18)
C3—C2—N1—C6	−1.3 (3)	C3A—C2A—N1A—C6A	−1.0 (3)

### Hydrogen-bond geometry (Å, º)

*Cg*5 and *Cg*6 are the centroids of the C14*A*–C19*A* and C20*A*–C25*A* phenyl rings of molecule II, respectively.

**Table:**

*D*—H···*A*	*D*—H	H···*A*	*D*···*A*	*D*—H···*A*
O1—H1*A*···N1	0.93 (2)	1.93 (2)	2.853 (2)	169 (3)
O1—H1*B*···N7*A*^i^	0.89 (2)	2.33 (2)	3.163 (3)	156 (3)
O1*A*—H1*C*···N1*A*	1.04 (2)	1.78 (2)	2.811 (3)	174 (2)
O1*A*—H1*C*···N2*A*	1.04 (2)	2.61 (2)	3.174 (3)	114 (1)
O1*A*—H1*D*···N7^ii^	0.91 (2)	2.38 (2)	3.206 (3)	152 (2)
O1*A*—H1*D*···N13*A*	0.91 (2)	2.59 (2)	3.153 (3)	121 (2)
N2—H2*A*···O1	0.86 (3)	2.44 (3)	3.140 (3)	139 (2)
N2—H2*A*···O1^iii^	0.86 (3)	2.29 (3)	2.892 (3)	127 (2)
N2—H2*B*···N7*A*^iv^	0.87 (3)	2.41 (3)	3.209 (3)	151.7 (18)
N2*A*—H2*C*···O1*A*	0.87 (3)	2.48 (3)	3.174 (3)	137 (2)
N2*A*—H2*C*···O1*A*^v^	0.87 (3)	2.25 (3)	2.859 (3)	127 (3)
N2*A*—H2*D*···N7^iv^	0.85 (3)	2.42 (3)	3.205 (3)	154 (3)
N4—H4*A*···N13*A*^vi^	0.82 (3)	2.21 (3)	2.984 (3)	158 (3)
N4*A*—H4*C*···N13^vii^	0.84 (3)	2.16 (3)	2.930 (3)	152 (2)
C11—H11···N8*A*	0.95	2.59	3.453 (3)	151
C11*A*—H11*A*···N8^viii^	0.95	2.49	3.369 (3)	154
C21—H21···*Cg*6^ix^	0.95	2.91	3.653 (2)	136
C26*A*—H26*F*···*Cg*5	0.98	2.97	3.781 (2)	141

Symmetry codes: (i) *x*−1, *y*, *z*; (ii) *x*−1, *y*+1, *z*; (iii) −*x*+1, −*y*+1, −*z*+1; (iv) −*x*+2, −*y*+1, −*z*+1; (v) −*x*+1, −*y*+2, −*z*+1; (vi) *x*+1, *y*−1, *z*; (vii) *x*+1, *y*, *z*; (viii) *x*, *y*+1, *z*; (ix) −*x*+1, −*y*+1, −*z*.
